# Regulation of the orphan G-protein–coupled receptor GPRC5B by MLC1 and the cell adhesion molecule GlialCAM in megalencephalic leukoencephalopathy

**DOI:** 10.1016/j.jbc.2025.110987

**Published:** 2025-11-27

**Authors:** Guillem Pont-Espinós, Adrià Pla-Casillanis, Laura Ferigle, Marta Alonso-Gardón, Marc González-Subías, Xabier Elorza-Vidal, Héctor Gaitán-Peñas, Ekaitz Errasti-Murugarren, Andy Chevigne, Tania López-Hernández, Francisco Ciruela, Raúl Estévez

**Affiliations:** 1 Physiology Unit, Department of Physiological Sciences, School of Medicine and Health Sciences, Institute of Neurosciences, University of Barcelona, Neuroscience Program, Physiology and Pathology of the Functional Relationship Between Glia and Neurons-IDIBELL, L'Hospitalet de Llobregat, Spain; 2 Genetic Unit, Department of Physiological Sciences, School of Medicine and Health Sciences, Institute of Neurosciences, University of Barcelona, Neuroscience Program, Physiology and Pathology of the Functional Relationship Between Glia and Neurons-IDIBELL, L'Hospitalet de Llobregat, Spain; 3 Centro de Investigación en Red de Enfermedades Raras (CIBERER), Instituto de Salud Carlos III, Madrid, Spain; 4 Department of Infection and Immunity, Immuno-Pharmacology and Interactomics, Luxembourg Institute of Health (LIH), Esch-sur-Alzette, Luxembourg; 5 Pharmacology Unit, Department of Pathology and Experimental Therapeutics, School of Medicine and Health Sciences, Institute of Neurosciences, University of Barcelona, Neuropharmacology & Pain Group, Neuroscience Program, Bellvitge Institute for Biomedical Research, L'Hospitalet de Llobregat, Spain

**Keywords:** leukodystrophy, brain water homeostasis, G-protein–coupled receptors, protein network, MLC

## Abstract

Megalencephalic leukoencephalopathy with subcortical cyst (MLC) is a rare leukodystrophy primarily caused by mutations in two genes: MLC1, encoding a membrane protein of unknown function, and GlialCAM, a cell adhesion molecule. Although MLC1 has been implicated in downregulating signaling pathways, its molecular mechanisms remain elusive. Recently, the orphan G-protein–coupled receptor GPRC5B was identified as a novel interactor of both GlialCAM and MLC1, with dominant heterozygous mutations found in MLC patients, suggesting that GlialCAM and MLC1 may regulate cell signaling *via* GPRC5B. Here, we show that GPRC5B exhibits constitutive activity, which is inhibited by MLC1, likely through interference with GPRC5B oligomerization. Conversely, GlialCAM enhances β-arrestin 2 recruitment, leading to its own mislocalization from cell–cell junctions. MLC-associated GPRC5B mutants show enhanced maturation and increased stability at the plasma membrane, retain normal constitutive activity and responsiveness to MLC1 and GlialCAM but display increased affinity for GlialCAM and localize to cell–cell junctions in its presence. Notably, coexpression of GlialCAM with these mutants does not induce GlialCAM mislocalization. We propose a model in which finely tuned interactions among GPRC5B, GlialCAM, and MLC1 regulate receptor signaling. These findings provide the first biochemical evidence of GlialCAM and MLC1 modulating GPRC5B activity, suggesting a biochemical explanation for the gain-of-function phenotype observed in GPRC5B MLC mutants. Importantly, our work supports the potential of targeting GPRC5B as a therapeutic strategy in MLC.

Megalencephalic leukoencephalopathy with subcortical cyst (MLC) is a rare leukodystrophy primarily characterized by vacuolization of myelin and astrocytes (1). Clinically, MLC manifests with early onset macrocephaly, progressive motor impairment, including ataxia and spasticity, epileptic seizures, and cognitive decline (2). Even minor head trauma or common infections can exacerbate symptoms, triggering acute motor deterioration, seizures, status epilepticus, or prolonged states of unconsciousness (3, 4, 5). Currently, no curative therapies exist; only symptomatic support is available.

The majority of MLC cases are linked to mutations in the *MLC1* gene (MIM no.: 605908; type MLC1) (6), whereas a smaller proportion is associated with mutations in *GLIALCAM* (MIM no.: 611642; type MLC2) (7). *MLC1* encodes a membrane protein predominantly expressed in astrocytic perivascular end feet and in Bergmann glia of the cerebellum (8, 9, 10). GlialCAM, an immunoglobulin-like cell adhesion molecule, acts as a chaperone and stabilizer of MLC1 at cell–cell junctions. Together, MLC1 and GlialCAM form a functional complex that regulates astrocyte volume homeostasis and intercellular communication, particularly under osmotic stress conditions (11, 12, 13, 14). Disruption of this complex because of pathogenic variants leads to protein mislocalization, impaired ion channel regulation, and astrocytic dysfunction, which are central to MLC pathophysiology.

This complex modulates the activity and localization of several key astrocytic proteins, including the chloride channel 2 (ClC-2) (15), the gap junction protein Cx43 (connexin 43) (16, 17, 18), the volume-regulated anion channel (19, 20, 21, 22), and the Na^+^/K^+^-ATPase pump (23, 24). Recent studies suggest that the GlialCAM–MLC1 complex also influences signaling pathways, including those mediated *via* extracellular signal–regulated kinase (ERK) and NF-κB (14, 16, 21, 25). Notably, MLC1 overexpression suppresses inflammatory signaling *via* phosphorylated ERK and pNF-κB, whereas *Mlc1*-KO astrocytes exhibit enhanced activation of these pathways (14, 21), although the precise molecular mechanisms remain unclear.

To gain further insights into these regulatory roles, we performed immunoaffinity experiments using newly developed antibodies against GlialCAM and MLC1, along with KO models. Coupled with a membrane yeast two-hybrid screening, these experiments revealed novel interacting partners, including G-protein–coupled receptors (GPCRs) and, importantly, the orphan receptor GPRC5B, which interacts directly with both GlialCAM and MLC1 (26). GPRC5B belongs to the class C family of GPCRs, a group characterized by large extracellular domains, constitutive activity, and the ability to form homodimers or heterodimers (27). Although GPRC5B lacks a known endogenous ligand, its structural classification suggests potential for complex regulatory interactions and noncanonical signaling mechanisms.

In nonastrocytic cells, GPRC5B overexpression activates inflammatory signaling pathways, including ERK and NF-κB, through interactions with the Src-family tyrosine kinase Fyn (28, 29, 30). This interaction depends on specific tyrosine residues in the cytoplasmatic tail of GPRC5B. In particular, a point mutation in mouse GPRC5B (Y383F) abolishes Fyn binding and downstream NF-κB activation in adipocytes, highlighting the functional importance of this residue in signal transduction. This constitutive activity of GPRC5B promotes expression of inflammatory mediators like intercellular adhesion molecule 1 and vascular cell adhesion molecule 1 (VCAM-1). GPRC5B has also been reported to interact with sphingomyelin synthase 2 (31) and other GPCRs, including prostaglandin receptors (32). However, GPRC5B signaling mechanisms in astrocytes remain poorly understood.

Recently, dominant *GPRC5B* mutations (MIM no.: 605948) have been identified in three unrelated MLC patients (type MLC3) (33, 34). Patient-derived lymphocytes exhibited elevated GPRC5B expression, and overexpression in U251 astrocytoma cells demonstrated increased volume-regulated anion channel currents, an effect preserved in GPRC5B variants harboring these mutations (34). However, the molecular pathology underlying GPRC5B-related MLC remains unresolved.

In this study, we aim to clarify the functional relationship among GlialCAM, MLC1, and GPRC5B and to gain insights into the molecular pathogenesis of MLC3 mutations in GPRC5B. Our findings offer new molecular insights into the modulation of GPRC5B signaling by MLC1 and GlialCAM, specifically in regulating the constitutive signaling activity of GPRC5B, and propose a mechanistic model explaining how this protein complex may contribute to the pathogenesis of GPRC5B and MLC1-associated leukodystrophy.

## Results

### GPRC5B exhibits constitutive signaling activity in living cells

*GPRC5B* was recently identified as the third gene implicated in MLC (33). It belongs to class C of the GPCR family, a group characterized by constitutive activity and the capacity to form both homodimers and heterodimers (27).

**Figure 1:**
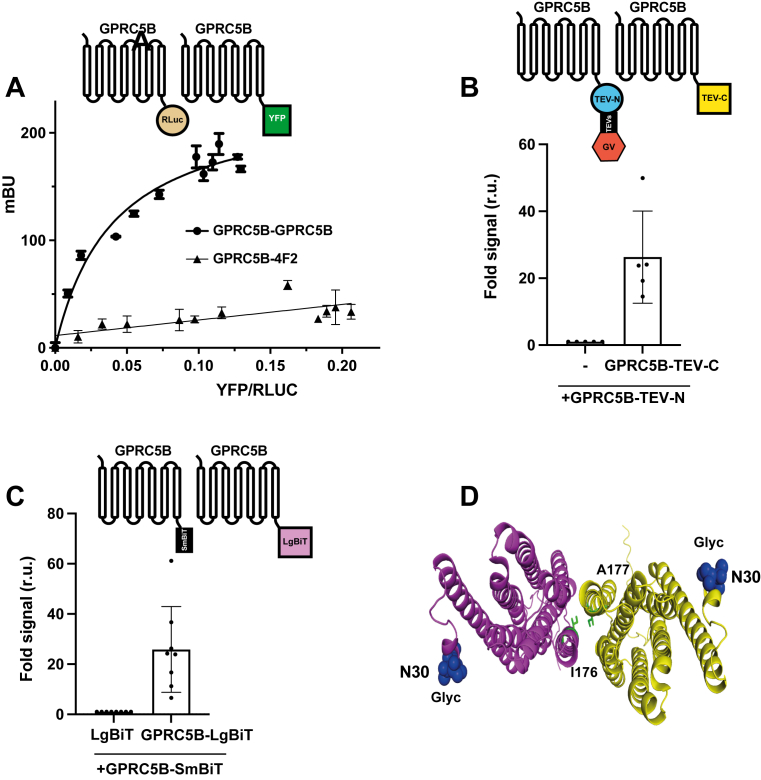
**GPRC5B forms homo-oligomers, and MLC3 mutations localize at the dimer interface**. *A*, bioluminescence resonance energy transfer (BRET) assay showing energy transfer between GPRC5B-RLuc and GPRC5B-YFP fusion proteins, indicating homo-oligomerization. GPRC5B-4F2 was used as a negative control. Another independent experiment gave similar results. *B*, split-TEV protease complementation assay demonstrating GPRC5B–GPRC5B interaction. Coexpression of GPRC5B-TEV-N and GPRC5B-TEV-C results in a significant increase in luciferase activity compared with controls. *C*, NanoLuc luciferase complementation assay confirming GPRC5B homodimerization. Coexpression of GPRC5B-LgBiT and GPRC5B-SmBiT leads to a robust luminescent signal, whereas controls show minimal activity. Schemes in *A*, *B*, and *C* show the type of experiments performed. *D*, structural model of a GPRC5B dimer generated using the AlphaFold 3D web server. The positions of the MLC3-associated mutations I176dup and A177dup are located at the predicted dimer interface within the fourth transmembrane domain, suggesting a potential dominant effect on receptor oligomerization. The localization of a putative glycosylation site at the N terminus is also indicated. LgBiT, large part of NanoLuc luciferase; MLC, megalencephalic leukoencephalopathy with subcortical cyst; SmBiT, small part of NanoLuc luciferase; TEV, tobacco etch virus.

To investigate whether GPRC5B can homo-oligomerize, we employed three complementary experimental approaches: bioluminescence resonance energy transfer (BRET), split-tobacco etch virus (TEV) protease complementation, and NanoLuc luciferase complementation (Fig. 1, *A*–*C*). All three assays consistently demonstrated that GPRC5B is capable of self-interaction.

Given the ability of GPRC5B to form homo-oligomers, we performed structural modeling of a GPRC5B dimer using the AlphaFold 3D web server. Interestingly, this model revealed that the two *GPRC5B* MLC3 variants (33), I176dup and A177dup, which are located within the fourth transmembrane domain, are positioned at the dimer interface (Fig. 1*D*). This finding suggests that the mutations may exert dominant effects, consistent with their mode of inheritance.

Next, we evaluated whether GPRC5B exhibits basal (ligand-independent) signaling activity. Given that astrocytes are the principal cell types affected in MLC, we first examined GPRC5B signaling in primary astrocytes. Prior studies showed that in vascular tissues, GPRC5B enhances NF-κB activation *via* its interaction with the Src-family kinase Fyn, leading to increased expression of adhesion molecules, such as VCAM-1 (28). Notably, a point mutation (Y383F) in the cytoplasmatic tail of mouse GPRC5B (Y376 in human GPRC5B) disrupts this interaction and abolishes downstream NF-κB activation in adipocytes (29). To assess whether this mechanism is conserved in astrocytes, we overexpressed WT (GPRC5B^WT^) or mutant (GPRC5B^Y376F^) forms of the human receptor *via* adenoviral delivery in primary rat astrocytes.

**Figure 2:**
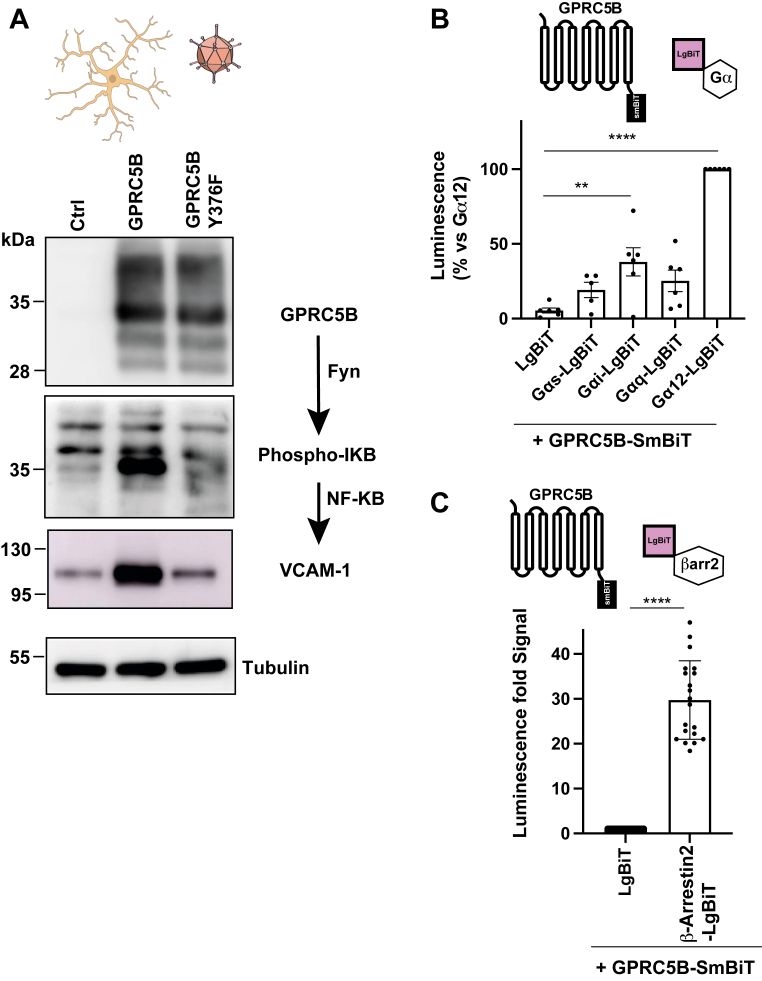
**Constitutive activity of GPRC5B in primary astrocytes and HEK293T cells**. *A*, Western blot analysis of primary rat astrocytes *left* untransduced (Ctrl) or transduced with adenoviral particles expressing WT human GPRC5B (5B) or the Y376F variant (MOI = 2). The scheme of the experiment is shown in the *top*. The mutation Y376F in human GPRC5B corresponds to Y383F in mouse GPRC5B. Blots probed for GPRC5B, phospho-IKB, VCAM-1, and tubulin (loading control). Representative of three experiments. The schematic illustrates the GPRC5B–Fyn–NF-κB –VCAM-1 signaling pathway. *B*, quantification of GPRC5B–SmBiT coupling to different G_α_ subunits or LgBiT (negative control) *via* NanoBiT assays in HEK293T cells. At the *top*, we indicate a scheme of the experiment. The G_α12/13_ group was set at 100%. The data represent five to six independent experiments. Statistical analysis by one-way ANOVA with Tukey’s post hoc test. ∗∗∗∗*p* < 0.0001, ∗∗*p* < 0.01 *versus* GPRC5B–SmBiT + LgBiT. *C*, NanoBIT signal showing GPRC5B–SmBiT interaction with β-arrestin 2–LgBIT. At the *top*, we indicate a scheme of the experiment. The data from 20 experiments. ∗∗∗∗*p* < 0.0001 *versus* GPRC5B–SmBiT + LgBiT. NanoLuc/RLuc ratio is shown. Mean ± SD. HEK293T, human embryonic kidney 293T cell line; LgBiT, large part of NanoLuc luciferase; MOI, multiplicity of infection; SmBiT, small part of NanoLuc luciferase; VCAM-1, vascular cell adhesion molecule 1.

As illustrated in Figure 2*A*, overexpression of GPRC5B^WT^ markedly increased IkB phosphorylation and upregulated VCAM-1 expression. In contrast, expression of GPRC5B^Y376F^ failed to elicit either response, consistent with Fyn-dependent signaling activity. These findings confirm that GPRC5B activates inflammatory pathways in astrocytes *via* a conserved mechanism and that VCAM-1 induction is not because of generalized cellular stress.

In parallel, we explored additional G-protein–dependent signaling routes. Prior evidence indicated that GPRC5B can activate β-catenin signaling through coupling with Gα_12/13_ proteins in the developing neocortex (35). To systematically assess G-protein engagement, we employed a NanoBiT-based assay using human embryonic kidney 293T (HEK293T) cells (36) transiently expressing GPRC5B fused to small BiT (SmBiT) together with mini versions of heterotrimeric G proteins (mini-G_αi/o_, G_αs_, G_αq/11_, and G_α12_) fused to large BiT (LgBiT). This system enables quantification of constitutive receptor coupling based on luminescence signal output (37). Importantly, we also quantified mini-G protein levels using HiBiT complementation (Supplementary Fig. S1*A*) to rule out the possibility that the observed signal increases were due to differences in construct expression levels.

As shown in Figure 2*B*, GPRC5B exhibited the strongest constitutive interaction with G_α12_, consistent with previous reports (35). A weaker, yet significant, association was also observed with the G_αi/o_ subfamily (Fig. 2*B*). These findings align with the established notion that orphan GPCRs frequently display functional promiscuity by coupling to multiple G-protein families (38).

In addition, we evaluated the ability of GPRC5B to engage β-arrestin 2, a common signaling and internalization effector of GPCRs. Coexpression of GPRC5B with β-arrestin 2-LgBiT yielded a significant NanoBiT signal (Fig. 2*C*), indicating constitutive interaction. This supports previous observations that many GPCRs form stable complexes with β-arrestin even in the absence of exogenous ligand (39).

Taken together, these results confirm that GPRC5B is a constitutively active receptor, capable of engaging multiple signaling partners, including Fyn, G_α12_ proteins, and β-arrestin 2. This activity is preserved in astrocytes and HEK293T cells and is likely driven by GPRC5B expression levels, as commonly observed for orphan GPCRs (40).

### Modulatory effects of MLC1 and GlialCAM on GPRC5B signaling dynamics

Our previous work demonstrated that GPRC5B directly interacts with MLC1 and GlialCAM (26). Furthermore, MLC1 has been related to signal transduction (25), suggesting a potential functional interplay among these proteins. To explore this hypothesis, we next investigated whether coexpression of MLC1 or GlialCAM with GPRC5B could influence its downstream signaling. Initial experiments using adenoviral-mediated coexpression in primary astrocytes revealed a nonspecific upregulation of VCAM-1, likely caused by toxicity because of high levels of virus, which rendered further analysis in this system unfeasible. Therefore, we turned to HEK293T cells, transfected with NanoBiT constructs, to systematically evaluate the modulatory effects of MLC1 and GlialCAM.

**Figure 3:**
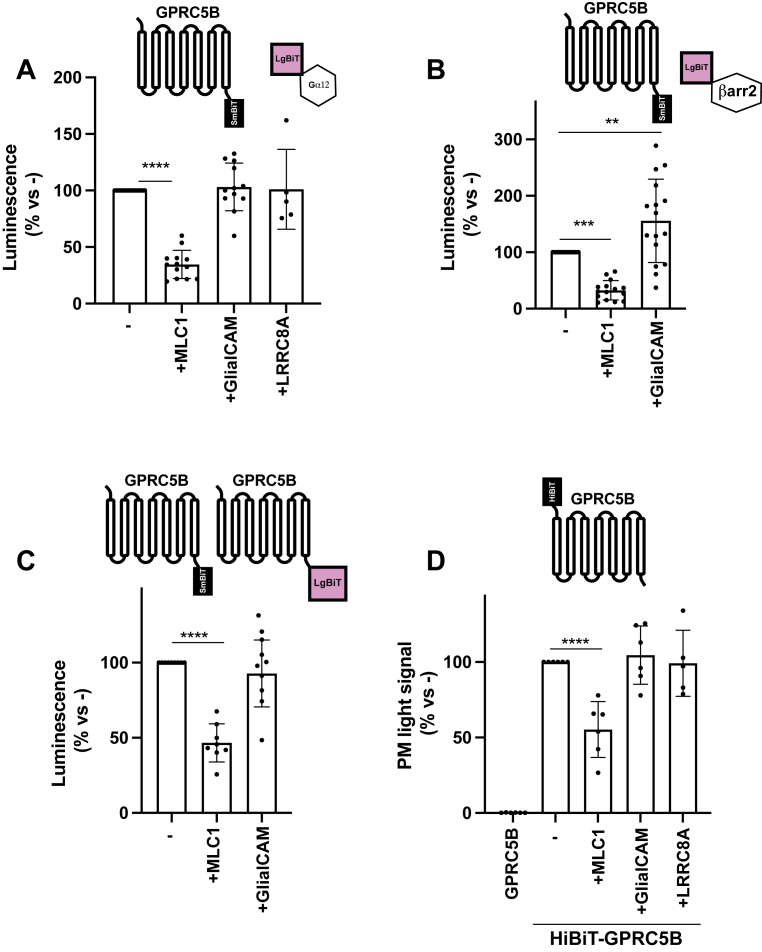
**Modulation of GPRC5B signaling and surface expression by MLC1 and GlialCAM**. *A*, nanoBiT assay showing the effect of MLC1, GlialCAM, or LRRC8A on the interaction between GPRC5B–SmBiT and Gα12–LgBiT in HEK293T cells. Coexpression of MLC1 significantly reduced GPRC5B–Gα12 coupling, whereas GlialCAM and LRRC8A had no effect. A schematic above the graph illustrates the NanoBiT-based interaction setup. Data represent 5 to 13 independent experiments. ∗∗∗∗*p* < 0.0001 *versus* GPRC5B + Gα12. *B*, nanoBiT assay assessing the interaction between GPRC5B–SmBiT and β-arrestin 2–LgBiT in the presence of MLC1 or GlialCAM. MLC1 reduced β-arrestin 2 recruitment, whereas GlialCAM significantly enhanced it. A schematic above the graph depicts the NanoBiT interaction. Data from 16 independent experiments. ∗∗*p* < 0.01; ∗∗∗*p* < 0.005. *C*, nanoBiT-based homodimerization assay showing that MLC1 coexpression significantly decreases GPRC5B–GPRC5B interaction, whereas GlialCAM has no detectable effect. The schematic above the graph shows the NanoBiT configuration used to monitor dimerization. Data from four to five experiments. ∗∗∗*p* < 0.001 *versus* GPRC5B–SmBiT + GPRC5B–LgBiT. *D*, quantification of GPRC5B surface expression using an HiBiT-tagged extracellular construct. Coexpression of MLC1 markedly reduced plasma membrane localization of GPRC5B, whereas GlialCAM and LRRC8A had no significant impact. A schematic above the graph illustrates the HiBiT-tagged GPRC5B construct used to monitor surface expression. Data are presented as a percentage of control (GPRC5B alone). Mean ± SD. GlialCAM, glial cell adhesion molecule; HEK293T, human embryonic kidney 293T cell line; MLC, megalencephalic leukoencephalopathy with subcortical cyst; SmBiT, small part of NanoLuc luciferase.

GPRC5B coupling to G_α12_ protein was assessed in the presence of MLC1, GlialCAM, or LRRC8A (as a specificity control). As shown in Figure 3*A*, coexpression of MLC1 significantly diminished the GPRC5B–G_α12_ interaction, whereas GlialCAM and LRRC8A had no effect. Importantly, Western blot analysis confirmed comparable expression of GPRC5B–smBiT across all conditions (Supplementary Fig. S1*B*), ruling out receptor downregulation as the cause of reduced signaling.

We next examined the interaction of GPRC5B with β-arrestin 2 using the same setup. Mirroring the G_α12_ findings, MLC1 reduced the GPRC5B–β-arrestin 2 interaction (Fig. 3*B*). By contrast, GlialCAM significantly enhanced this association. As a control, Western blot analysis confirmed comparable expression levels of GPRC5B–smBiT across all experimental conditions (Supplementary Fig. S1*C*), thereby excluding receptor downregulation as the underlying cause of reduced signaling. These observations suggest that GlialCAM preferentially biases GPRC5B signaling toward β-arrestin 2 recruitment, potentially through complex formation, as previously reported for other GPCR heteromers (41, 42).

Given that class C GPCRs often require dimerization for surface expression and functional signaling (43, 44), and considering that GPRC5B forms homo-oligomers (Fig. 1), we hypothesized that MLC1 and GlialCAM might modulate GPRC5B homodimerization. Using a NanoBiT-based assay to monitor receptor oligomerization, we found that coexpression of MLC1 significantly reduced GPRC5B homodimerization, whereas GlialCAM had no detectable effect (Fig. 3*C*). As a control, quantification of the expression levels of GPRC5B–LgBiT across all conditions revealed similar levels (Supplementary Fig. S1*D*), ruling out receptor downregulation as a confounding factor.

To further investigate this effect, we assessed GPRC5B surface expression using an extracellularly tagged HiBiT–GPRC5B construct (Fig. 3*D*). Consistent with the oligomerization data, MLC1 coexpression led to a marked reduction in GPRC5B surface expression, whereas GlialCAM and LRRC8A had no significant impact.

Taken together, these findings suggest that MLC1 interferes with GPRC5B homodimerization and that this disruption may underlie the reduced surface expression of the receptor. This is consistent with the notion that dimerization is a prerequisite for membrane trafficking of class C GPCRs and supports a model in which MLC1 modulates GPRC5B function by impairing its oligomerization and subsequent localization to the plasma membrane. Altogether, these data indicate that MLC1 suppresses both G-protein–dependent and β-arrestin 2–dependent GPRC5B signaling, likely *via* disruption of receptor dimerization, whereas GlialCAM selectively promotes β-arrestin 2 engagement.

### GPRC5B alters the subcellular distribution of GlialCAM

Considering that GlialCAM enhances GPRC5B–β-arrestin 2 interaction, a process often linked with receptor internalization (39), we analyzed the subcellular localization of both proteins when expressed individually or together in HeLa cells.

**Figure 4:**
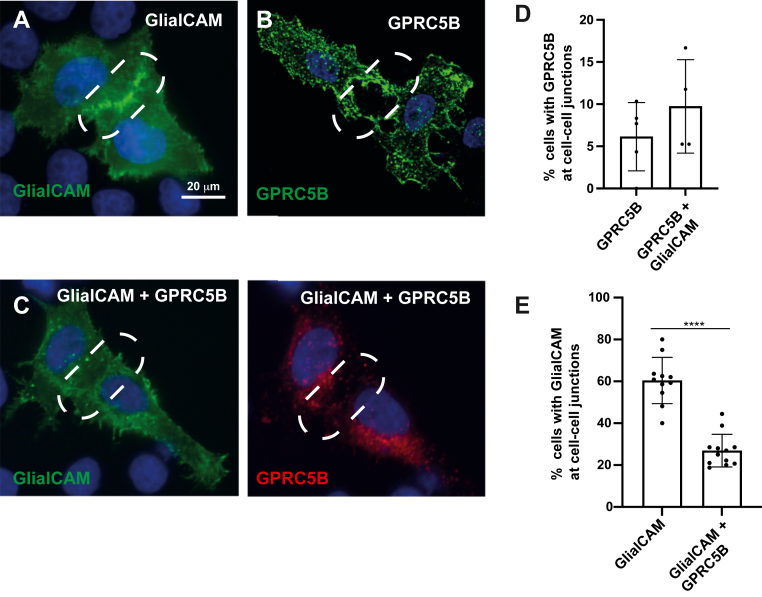
**GPRC5B alters GlialCAM subcellular localization**. *A*–*C*, immunofluorescence of HeLa cells transfected with GlialCAM-3xFLAG (*A*), GPRC5B (*B*), or both (*C*). GlialCAM alone localizes to cell–cell junctions, whereas GPRC5B shows diffuse distribution. *Dashed lines* indicate cell–cell junctions. Coexpression reduces junctional GlialCAM. The scale bar represents 20 μm. *D* and *E*, quantification of junctional enrichment of GPRC5B (*D*) and GlialCAM (*E*), with or without coexpression from 4 to 12 experiments, 30 to 50 pairs of cells/condition per experiment. ∗∗*p* < 0.01, ∗∗∗∗*p* < 0.0001 *versus* GlialCAM alone. Mean ± SD. GlialCAM, glial cell adhesion molecule.

Consistent with previous reports (7, 9, 15, 18, 45, 46, 47), GlialCAM localized predominantly at cell–cell junctions (Fig. 4, *A* and *E*), whereas GPRC5B displayed a diffuse plasma membrane distribution and was largely excluded from junctions when expressed alone (Fig. 4, *B* and *D*). Upon coexpression, GPRC5B remained only weakly detectable at junctions (Fig. 4, *C* and *D*), whereas GlialCAM’s junctional localization was markedly reduced (Fig. 4, *C* and *E*).

These findings suggest that GPRC5B can displace GlialCAM from intercellular contacts, potentially by altering its membrane trafficking or localization mechanisms. Considering previous results (Fig. 3*B*), this mislocalization of GlialCAM may be a consequence of β-arrestin 2 recruitment to GPRC5B, which could trigger internalization or redistribution of associated membrane proteins.

### Biochemical characterization of GPRC5B mutants identified in MLC type 3

To further dissect the relation between these three proteins in the pathogenesis mechanisms of MLC, we characterized two GPRC5B mutations associated with MLC type 3 (I176dup and A177dup).

**Figure 5:**
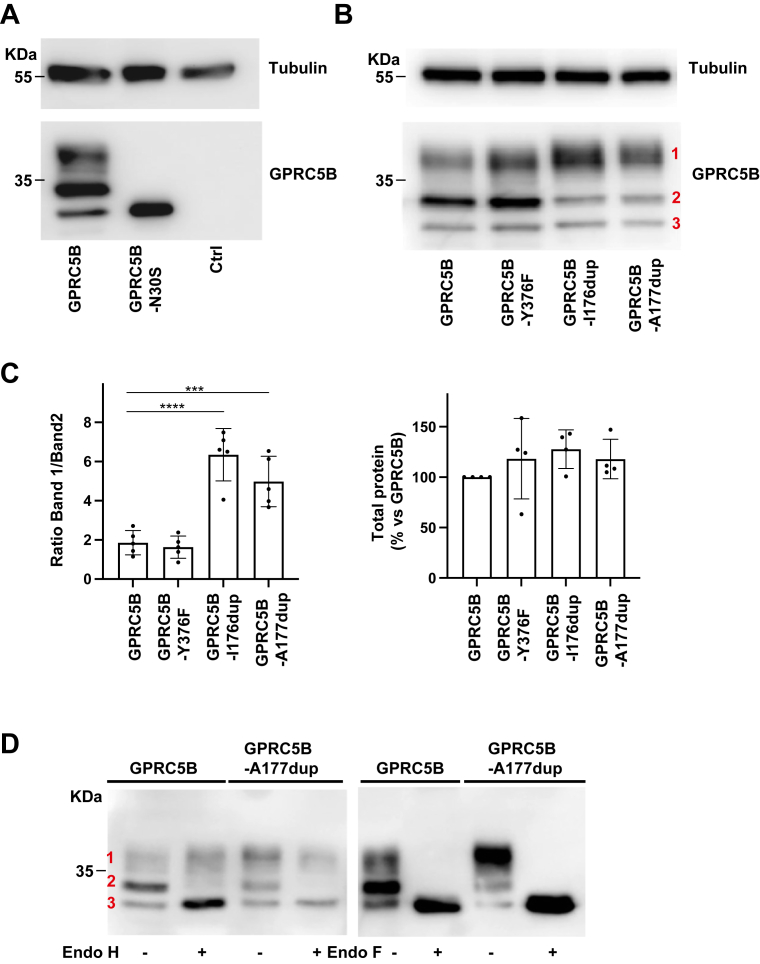
**Biochemical characterization of GPRC5B MLC3 mutants**. *A*, Western blot analysis of HEK293T cells transfected with WT GPRC5B or the N30S mutant, which disrupts the predicted N-linked glycosylation site at Asn30. The N30S variant shows a single lower–molecular-weight band, confirming glycosylation at this position. Tubulin was used as a loading control. *B*, Western blot analysis of cells expressing WT GPRC5B, the control Y376F variant, and the MLC3-associated mutants I176dup and A177dup. All constructs show three bands, but the mutants display altered band distribution. Tubulin served as a loading control. *C*, quantification of the ratio between the most glycosylated (band 1) and intermediate (band 2) forms of GPRC5B. Both I176dup and A177dup mutants show a significantly increased band 1/band 2 ratio compared with WT. Total GPRC5B protein levels (sum of all bands) remain unchanged. The data represent 5 independent experiments. ∗∗∗*p* < 0.001; ∗∗∗∗*p* < 0.0001 *versus* WT. *D*, glycosylation sensitivity of WT and A177dup GPRC5B assessed by enzymatic digestion with endo H (removes high-mannose glycans) and endo F (cleaves all N-linked glycans). Endo H treatment selectively reduces band 2, whereas endo F affects both bands 1 and 2, confirming the presence of complex glycosylation and enhanced maturation in the mutant receptor. HEK293T, human embryonic kidney 293T cell line; MLC, megalencephalic leukoencephalopathy with subcortical cyst.

Unlike other class C GPCRs (27), GPRC5B lacks a canonical ligand-binding domain at its N terminus. Sequence analysis predicted a potential N-linked glycosylation site at asparagine 30 within this region (Fig. 1*D*). In agreement with GPRC5B being glycosylated, Western blot analysis of WT GPRC5B revealed three distinct bands (Fig. 5*A*), likely reflecting glycosylation states. A GPRC5B variant in which Asn30 was substituted by Ser showed only the lower–molecular-weight species, confirming that glycosylation occurs at this position.

Expression of the two MLC3 GPRC5B mutants similarly resulted in three bands but with altered distribution: an increase in the most heavily glycosylated species (band 1) and a decrease in the intermediate form (band 2), whereas the nonglycosylated band (band 3) remained unchanged (Fig. 5*B*). Quantification of 5 independent experiments confirmed a significantly higher band 1/band 2 ratio in the mutants (Fig. 5*C*, *left*). Total protein levels, considering all three bands together, were unchanged (Fig. 5*C*, *right*). These results suggest that the mutants display enhanced post-translational maturation.

To further substantiate this hypothesis, protein lysates were treated with endo H (which removes high-mannose glycans) or endo F (which cleaves all N-glycans). Endo H selectively reduced the size of band 2, whereas endo F affected both bands 1 and 2 (Fig. 5*D*), indicating that the MLC3 mutants accumulate higher levels of complex glycosylated, and likely more stable, receptor species (48, 49).

**Figure 6:**
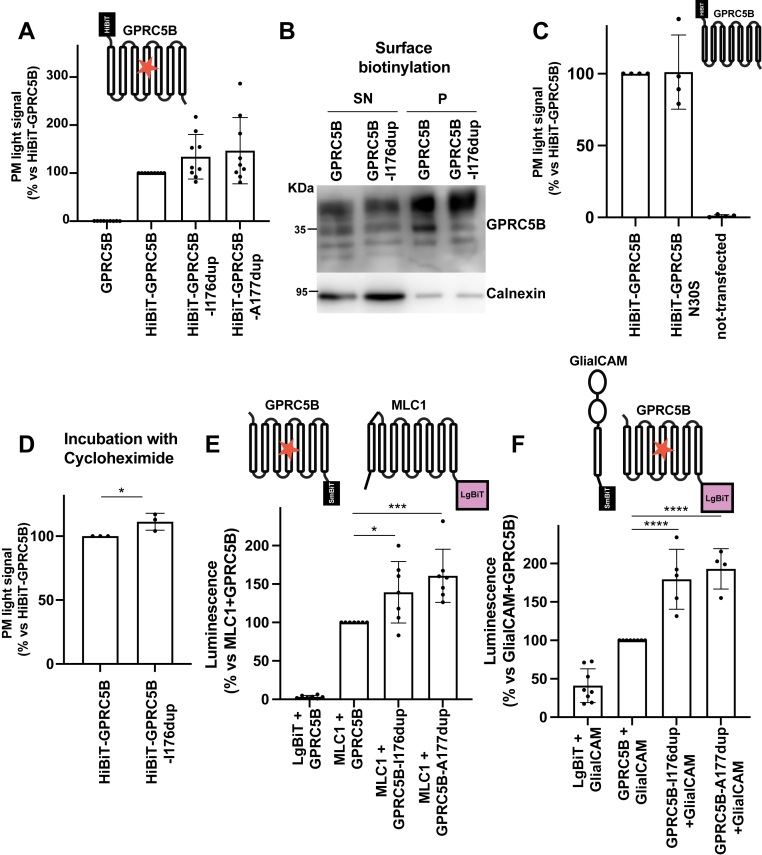
**Plasma membrane expression and interaction profile of GPRC5B MLC3 mutants**. *A*, surface expression of HiBiT-tagged WT and mutant GPRC5B (I176dup and A177dup) in HEK293T cells, measured by NanoLuc activity. All constructs showed comparable membrane localization. Data from eight independent experiments. *B*, surface biotinylation and streptavidin pulldown assay confirming membrane localization of WT and I176dup GPRC5B. Calnexin was used as a marker for membrane enrichment. Representative blot from three independent experiments. *C*, surface expression of HiBiT-tagged WT and N30S GPRC5B, showing that the nonglycosylated N30S variant reaches the plasma membrane similarly to the WT. Data from four experiments. *D*, receptor stability assessed by cycloheximide (CHX) chase assay. After 8 h of CHX treatment, the I176dup mutant showed a slight but significant increase in stability compared with WT. ∗*p* < 0.05. *E*, nanoBiT assay showing interaction between GPRC5B (WT or MLC3 mutants) and MLC1. The scheme above depicts the NanoBiT interaction configurations. Both I176dup and A177dup mutants exhibited mildly enhanced interaction with MLC1. Data from four to eight independent experiments. ∗*p* < 0.05; ∗∗∗*p* < 0.005; ∗∗∗∗*p* < 0.0001 *versus* WT. *F*, nanoBiT assay assessing interaction between GPRC5B variants and GlialCAM. The scheme above depicts the NanoBiT interaction configurations. Both MLC3 mutants showed significantly increased interaction with GlialCAM compared with WT. Data from four to eight independent experiments. ∗∗∗*p* < 0.005; ∗∗∗∗*p* < 0.0001 *versus* WT. GlialCAM, glial cell adhesion molecule; HEK293T, human embryonic kidney 293T cell line; MLC, megalencephalic leukoencephalopathy with subcortical cyst; NT, nontransfected control; P, streptavidin pellet; SN, supernatant.

Surface expression levels of the WT and mutant GPRC5B were evaluated using extracellular HiBiT tagging (Fig. 6*A*) and biotinylation assays (Fig. 6*B*), both of which revealed comparable membrane localization. All three glycosylated forms were present at the surface (Fig. 6*B*), and the nonglycosylated N30S variant also reached the membrane as the WT GPRC5B (Fig. 6*C*). Cycloheximide chase experiments performed in one mutant demonstrated slightly increased stability at the plasma membrane (Fig. 6*D*). Therefore, these experiments suggest that mutants may be more stable, which could explain their more mature glycosylation and progressive accumulation in the membrane over time. This is consistent with *in vivo* data, which showed higher levels of mutant GPRC5B protein in lymphoblasts from MLC3 patients (34).

We also examined the interaction of these GPRC5B mutants with GlialCAM and MLC1 using NanoBIT assays. These experiments showed that both MLC3 mutants displayed mildly enhanced interaction with MLC1 (Fig. 6*E*) and more pronounced interaction with GlialCAM (Fig. 6*F*), suggesting that the mutations might subtly alter receptor-binding preferences.

### Functional signaling assessment of GPRC5B MLC3 mutants

Therefore, our experiments indicated that MLC3 mutants exhibit enhanced maturation, increased plasma membrane stability, and a stronger interaction with GlialCAM, suggesting that the mutations cause a gain of function regarding receptor stability and membrane localization.

**Figure 7:**
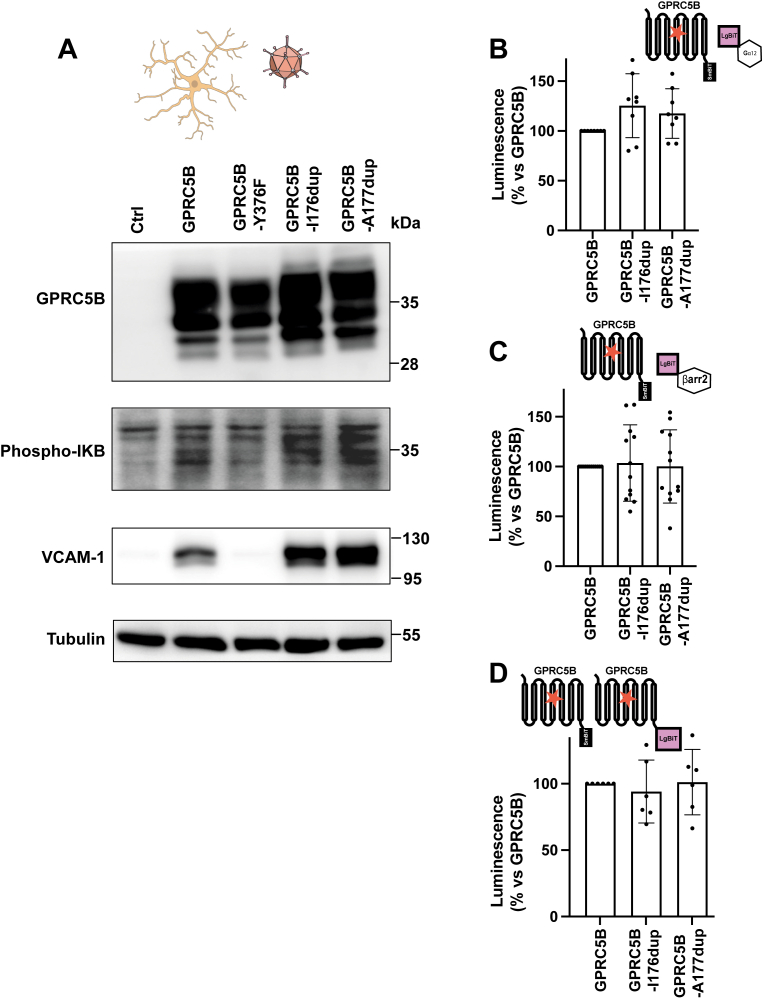
**Functional activity of GPRC5B MLC3 mutants**. *A*, Western blot analysis of primary rat astrocytes transduced with WT GPRC5B, MLC3-associated mutants (I176dup and A177dup), or the signaling-deficient Y376F variant (negative control). Both MLC3 mutants induced increased phosphorylation of IκB and upregulation of VCAM-1 expression compared with WT. Tubulin was used as a loading control. A schematic above the blot illustrates the type of experiment performed. Representative of three independent experiments. *B* and *C*, nanoBiT assays in HEK293T cells quantifying the interaction of GPRC5B variants with Gα12–LgBiT (*B*) or β-arrestin 2–LgBiT (*C*). WT GPRC5B was set to 100%. Both MLC3 mutants showed similar coupling levels to Gα12 and β-arrestin 2 as the WT receptor. Schematics above each graph depict the NanoBiT interaction configurations. Data from 8 (*B*) and 12 (*C*) independent experiments. No significant differences were observed (*p* > 0.05). *D*, nanoBiT assay assessing homodimerization of WT and MLC3 mutant GPRC5B. Both I176dup and A177dup mutants exhibited homodimerization levels comparable to WT. A schematic above the graph illustrates the NanoBiT-based dimerization assay. Data from six independent experiments. No significant differences *versus* WT. LgBiT-only construct was used as a negative control. HEK293T, human embryonic kidney 293T cell line; LgBiT, large part of NanoLuc luciferase; MLC, megalencephalic leukoencephalopathy with subcortical cyst; VCAM-1, vascular cell adhesion molecule 1.

We then evaluated whether the MLC3-associated GPRC5B mutants affect the constitutive activity of GPRC5B. We first expressed both GPRC5B variants in primary astrocytes. Both variants induced IkB phosphorylation and upregulated VCAM-1 expression, similar to the WT receptor, but at higher levels, likely because of their increased expression (Fig. 7*A*).

**Figure 8:**
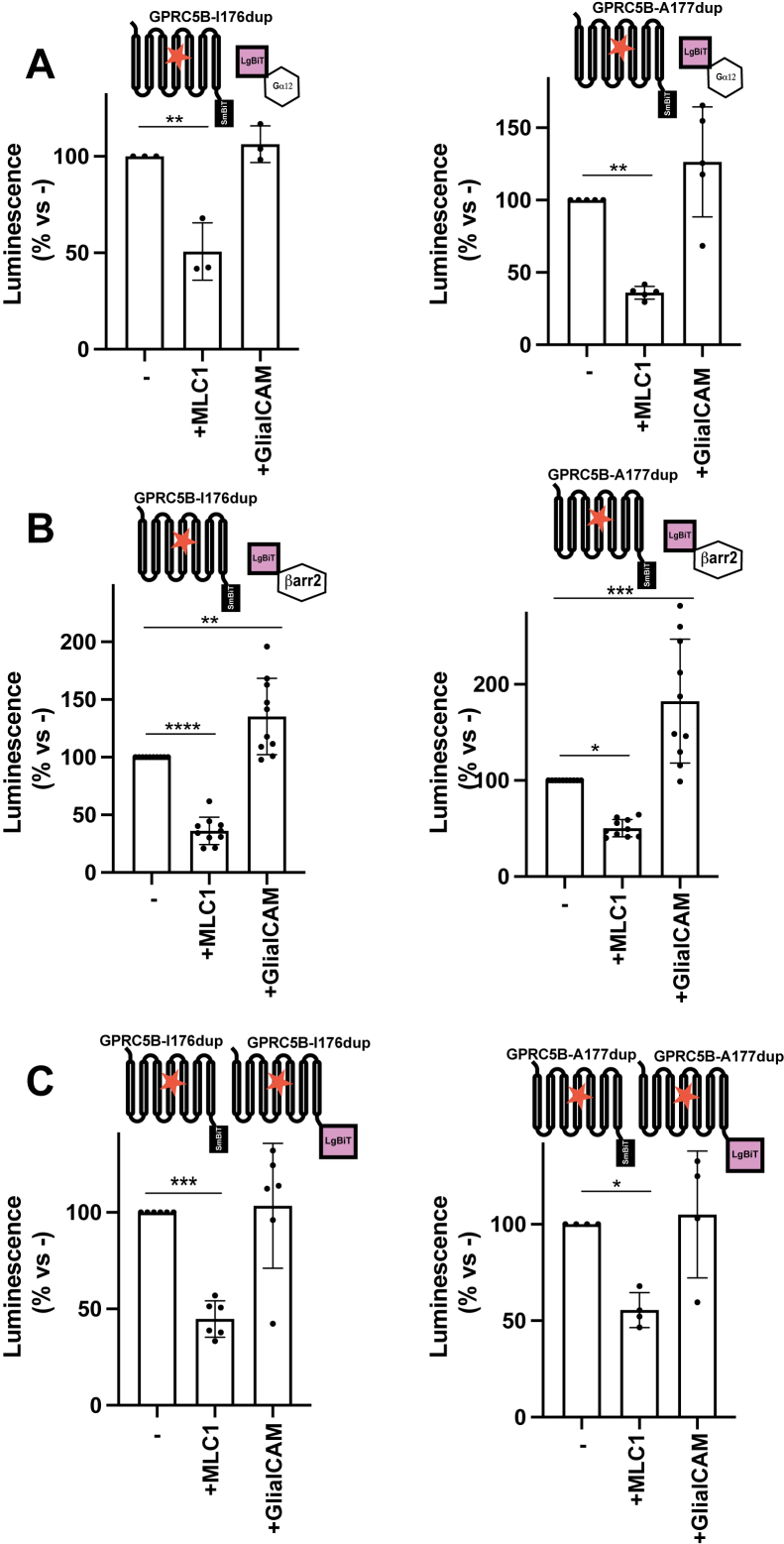
**Modulation of signaling and oligomerization of GPRC5B MLC3 mutants by MLC1 and GlialCAM**. A, nanoBiT assays assessing the effect of MLC1 and GlialCAM on the interaction between GPRC5B MLC3 mutants and Gα12. Coexpression of MLC1 significantly reduced Gα12 coupling for both I176dup (*left*) and A177dup (*right*) variants, whereas GlialCAM had no effect. Schematics above each graph illustrate the NanoBiT assay configuration. Data from three to five independent experiments. ∗∗*p* < 0.01 *versus* mutant alone. *B*, nanoBiT assays evaluating β-arrestin 2 recruitment by GPRC5B MLC3 mutants in the presence of MLC1 or GlialCAM. MLC1 reduced β-arrestin 2 interaction, whereas GlialCAM significantly enhanced it for both I176dup (*left*) and A177dup (*right*) variants. Schematics above each graph depict the NanoBiT interaction setup. Data from 9 to 10 independent experiments. ∗*p* < 0.05; ∗∗*p* < 0.01; ∗∗∗*p* < 0.005; ∗∗∗∗*p* < 0.0001 *versus* mutant alone. *C*, nanoBiT-based homodimerization assay showing the effect of MLC1 and GlialCAM on oligomerization of GPRC5B MLC3 mutants. MLC1 significantly reduced dimerization of both I176dup and A177dup, whereas GlialCAM had no significant effect. Schematics above each graph represent the NanoBiT configuration used to monitor dimerization. Data from four to six independent experiments. ∗*p* < 0.05 *versus* mutant alone. All data are presented as mean ± SD. GlialCAM, glial cell adhesion molecule; MLC, megalencephalic leukoencephalopathy with subcortical cyst.

NanoBiT analyses in HEK293T cells revealed that both mutants also interact with G_α12_ and β-arrestin 2 (Fig. 7, *B* and *C*) and display homodimerization levels comparable to the WT receptor (Fig. 7*D*). Moreover, regulatory responses to MLC1 and GlialCAM were preserved: MLC1 inhibited G_α12_ and β-arrestin 2 binding, whereas GlialCAM enhanced the latter (Fig. 8, *A* and *B*). MLC1 also reduced oligomerization of the mutants, consistent with its effects on WT GPRC5B, whereas GlialCAM had no such effect (Fig. 8*C*). These data indicate that the mutant receptors maintain normal signaling properties and remain responsive to physiological modulators.

### Identification of a subtle trafficking phenotype in GPRC5B MLC3 mutants

**Figure 9:**
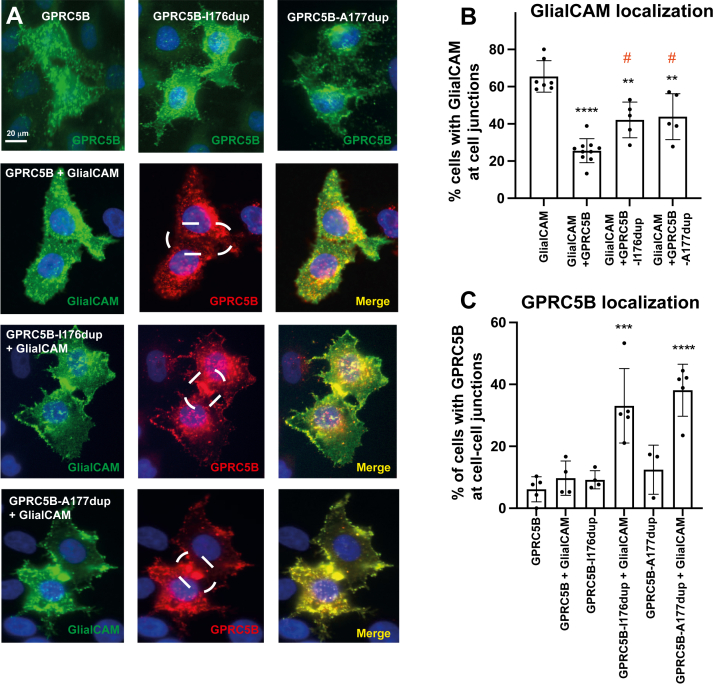
**Subcellular localization of GlialCAM and GPRC5B MLC3 mutants**. *A*, immunofluorescence analysis of HeLa cells transfected with WT or MLC3 mutant GPRC5B (I176dup, A177dup), either alone or coexpressed with GlialCAM-3xFLAG. GPRC5B was detected using a specific antibody against the transfected protein (*green* or *red*), and GlialCAM was detected using an anti-FLAG antibody. Colocalization at cell–cell junctions appears in *yellow* in merged images. Transfection conditions are indicated for each image. The scale bar represents 20 μm. *B*, quantification of GlialCAM localization at cell–cell junctions when expressed alone or coexpressed with WT or mutant GPRC5B. Coexpression with WT GPRC5B significantly reduced junctional localization of GlialCAM, whereas both MLC3 mutants preserved or enhanced junctional accumulation. ∗∗*p* < 0.01, ∗∗∗∗*p* < 0.0001 *versus* GlialCAM alone; #*p* < 0.05 *versus* GlialCAM + WT GPRC5B. *C*, quantification of GPRC5B enrichment at intercellular junctions in the absence or the presence of GlialCAM. Coexpression with GlialCAM significantly increased junctional localization of both I176dup and A177dup mutants compared with WT GPRC5B. ∗∗∗*p* < 0.005, ∗∗∗∗*p* < 0.0001 *versus* GPRC5B + GlialCAM. Data from >4 independent experiments. Mean ± SD is shown. GlialCAM, glial cell adhesion molecule; MLC, megalencephalic leukoencephalopathy with subcortical cyst.

We then assessed the effects of GPRC5B MLC3 mutants on GlialCAM localization. HeLa cells coexpressing GlialCAM with either GPRC5B mutant retained strong accumulation at cell junctions (Fig. 9, *A* and *B*), in contrast to the redistribution observed upon coexpression with WT GPRC5B (Figs. 4 and 9, *A* and *B*).

Notably, the mutants themselves exhibited increased enrichment at intercellular junctions when coexpressed with GlialCAM (Fig. 9, *A* and *C*), a pattern not observed with the WT receptor (Figs. 4 and 9, *A* and *B*). This altered distribution suggests that mutant GPRC5B displays enhanced retention at junctions through interactions with GlialCAM, potentially interfering with normal trafficking or complex turnover dynamics. Despite the increased recruitment of β-arrestin 2, this did not trigger GlialCAM internalization, hinting at modified responsiveness of the mutant receptor complexes.

## Discussion

Despite over 2 decades of investigation since the identification of the first MLC-associated gene (6), the molecular mechanisms driving MLC remain elusive (33). Our study provides compelling evidence for a regulatory network involving MLC1, GlialCAM, and the orphan GPCR GPRC5B, recently implicated in MLC type 3. We demonstrate that GPRC5B exhibits constitutive signaling activity and that leukodystrophy-associated GPRC5B mutations do not impair this activity but rather enhance receptor stability. Crucially, we show that MLC1 suppresses GPRC5B signaling, whereas GlialCAM facilitates its coupling to β-arrestin 2. This represents, to our knowledge, the first evidence of a functional interplay among MLC gene products converging on a shared signaling axis, opening new therapeutic avenues through modulation of GPRC5B activity.

Although GPRC5B remains an orphan receptor without an identified ligand (50), its ability to activate intracellular signaling in the absence of external stimuli enabled us to explore the modulatory effects of MLC1 and GlialCAM. Our results indicate that MLC1 negatively regulates GPRC5B constitutive activity by disrupting receptor oligomerization. Given that GPRC5B amplifies proinflammatory signals (29, 51, 52), these findings provide a mechanistic explanation for the elevated phosphorylated ERK and pNF-κB activity seen in *Mlc1*-KO astrocytes (21, 25). Taken together, we propose that MLC pathogenesis may be driven in part by unchecked GPRC5B signaling and that MLC3-associated mutations, which stabilize the receptor at the membrane, exacerbate this inflammatory drive.

**Figure 10:**
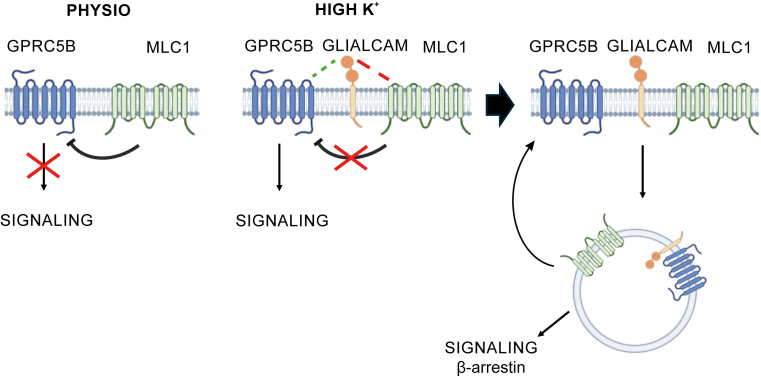
**Proposed model of GPRC5B regulation by GlialCAM and MLC1**. Schematic representation of the dynamic regulation of GPRC5B signaling by MLC1 and GlialCAM under different physiological conditions. *Left panel (physiological conditions)*, under resting conditions, MLC1 interacts with GPRC5B at the plasma membrane and suppresses its signaling activity. *Middle panel (depolarizing conditions)*, upon increased extracellular potassium (high K^+^), GlialCAM associates with MLC1 and/or GPRC5B, relieving MLC1-mediated inhibition and allowing GPRC5B activation. *Right panel (postactivation)*, following activation, GPRC5B and GlialCAM are internalized, leading to intracellular signaling events potentially mediated by β-arrestin 2 and/or GlialCAM. The precise subcellular compartment where this signaling occurs remains to be determined. GlialCAM, glial cell adhesion molecule; MLC, megalencephalic leukoencephalopathy with subcortical cyst.

To further explore the regulatory context of this signaling axis, we propose a model in which MLC complex activity is conditionally modulated by cellular depolarization (Fig. 10). MLC proteins have been implicated in buffering extracellular potassium during neuronal activity (53), suggesting that signaling through the MLC1–GlialCAM–GPRC5B complex may be triggered by depolarizing conditions (54). Several lines of evidence suggest that membrane potential may influence the distribution and interaction of proteins within the complex, including ClC-2 (54), GPRC5B (26), although the underlying mechanisms remain to be elucidated.

Of particular interest is the ability of GlialCAM to selectively enhance β-arrestin 2 recruitment to GPRC5B. β-arrestin binding is known to desensitize GPCRs, promote internalization, and initiate distinct intracellular signaling cascades (39). Although we do not define the precise cellular compartment where signaling occurs, our data support the existence of intracellular signaling of GPRC5B–GlialCAM complexes, potentially *via* β-arrestin 2 or GlialCAM itself (55) (Fig. 10). Future work will be needed to determine the precise localization of these events. Interestingly, previous studies have identified GPRC5B in exosomes derived from various cell types (56, 57). Moreover, recent work suggests that MLC1 and GlialCAM may also be present in extracellular vesicles (58), raising the possibility that components of the MLC signaling complex could participate in noncanonical intercellular communication pathways.

In parallel, our biochemical analysis revealed that MLC3 mutations enhance complex glycosylation of GPRC5B, a modification often associated with receptor stabilization at the plasma membrane (59, 60, 61). This likely prolongs receptor residency on the glial cell surface, amplifying inflammatory responses. In addition, MLC3-associated GPRC5B mutants form more stable complexes with GlialCAM that resist internalization, potentially disrupting β-arrestin 2 or GlialCAM signaling and impairing feedback regulation of surface activity. We further postulate that this imbalance could affect downstream targets responsible for brain homeostasis, such as aquaporin 4, a water channel also implicated in MLC (33). Reduced internal signaling may impair the phosphorylation or trafficking of such channels, contributing to the cerebral water accumulation observed in MLC patients, whereas heightened surface signaling could drive chronic inflammation.

Our findings also offer insight into the puzzling *in vivo* mislocalization of GlialCAM in *Mlc1*-KO mice (9), which contrasts with its normal localization in *Mlc1*-deficient astrocyte cultures. In depolarizing conditions, GlialCAM becomes internalized in the absence of MLC1 (10), suggesting that additional proteins, such as GPRC5B, regulate its trafficking. Indeed, GPRC5B–MLC1 and GPRC5B–GlialCAM interactions are enhanced during depolarization (26), and we hypothesize that GlialCAM internalization under these conditions is driven by GPRC5B association. In the absence of MLC1, GlialCAM may be internalized but retained intracellularly, disrupting its cell surface function.

Limitations of the study should be acknowledged. As with any complementation-based assay, the use of split-luciferase systems has inherent limitations, including potential sensitivity to tag orientation and steric effects. To mitigate these concerns, we implemented multiple controls and complementary approaches (BRET, Split-TEV, and surface expression assays), which collectively support the physiological relevance of the interactions studied. Furthermore, previous work has independently validated the association of GPRC5B with MLC1 and GlialCAM using orthogonal techniques, such as coimmunoprecipitation and BRET (26), and the interaction with Gα12 is consistent with prior functional evidence (35). While no method is entirely free of caveats, the convergence of these data strengthens confidence in our conclusions.

In conclusion, this study identifies GPRC5B signaling as a central axis in MLC pathogenesis and uncovers dynamic regulation by the astrocytic proteins MLC1 and GlialCAM. We show that MLC3-associated GPRC5B mutations shift receptor behavior by enhancing surface stability and altering downstream signaling. These findings support a unified model in which dysregulated GPRC5B activity, because of either loss of modulation by MLC1–GlialCAM or intrinsic receptor mutations, drives neuroinflammatory and homeostatic defects in MLC. Targeting this signaling pathway may offer a promising route for therapeutic intervention in this currently untreatable leukodystrophy.

## Experimental procedures

### Molecular biology

Plasmid constructs were generated using standard recombinant PCR techniques in combination with the Multisite Gateway Cloning System (Invitrogen). All insert constructs were verified by Sanger sequencing. Viral vectors were produced and purified following previously established protocols (26).

### Cell culture and transfection

HeLa and HEK293T cells were cultured in Dulbecco’s modified Eagle’s medium supplemented with 10% fetal bovine serum (Gibco), 1% glutamine, and 1% penicillin–streptomycin. Cells were maintained at 37°C in a humidified 5% CO_2_ atmosphere. Transient transfections were performed using Transfectin Lipid Reagent (Bio-Rad) following the manufacturer’s protocol. Experiments were conducted 24 to 48 h post-transfection. HEK293T cells were selected for interaction assays and Western blot experiments because of their high transfection efficiency. HeLa cells were used for immunofluorescence. HeLa cells were used for immunofluorescence localization studies. These cells are well suited for expressing multiple plasmids simultaneously and facilitate reliable imaging. In contrast, HEK293T cells tend to detach easily from coverslips, making them less suitable for microscopy-based assays. Importantly, we have a well-established history of using HeLa cells in previous studies involving GlialCAM, which provides a solid framework for interpreting the new experiments presented here.

### Split-nanoluciferase experiments

The LgBiT and SmBiT fragments of NanoLuc Luciferase (Promega) were amplified by PCR with glycine–serine linkers and cloned into N- or C-terminal gateway-compatible entry vectors. Mini-Gα constructs (Gα_i_, Gα_s_, Gα_q_, and mini-Gα_12/13_), each fused to LgBiT, were obtained from previously published studies (62, 63). A β-arrestin2–LgBiT construct was kindly provided by Dr K. Sahlholm (Karolinska Institute).

HEK293T cells were transiently transfected with SmBiT-tagged GPRC5B, MLC1, or GlialCAM together with the indicated LgBiT-tagged mini-Gα-LgBiT subunits or β-arrestin. The transfection control of pGK-Rluc was included in all conditions. Where indicated, pCDNA3.1-empty, pCDNA3.1-MLC1, or pCDNA3.1-GlialCAM vectors were also added. Cells were harvested in 1 ml of PBS, and 50 μl of the suspension was seeded into white, flat-bottom 96-well plates (Bio-Rad; 50,000 cells/cm^2^). NanoLuc and Rluc activities were measured using Promega reagents according to the manufacturer’s instructions. Luminescence was recorded with a CLARIOstar plate reader after 10 min of incubation, and the normalized NanoLuc signal (normalized light unit/relative light unit) was used for analysis.

Quantification of G proteins fused to LgBiT was carried out using the Nano-Glo HiBiT Lytic detection system following the supplier’s instructions. The HiBiT control protein (N3010; Promega) was used in place of the LgBiT protein provided in the kit.

### Split-TEV experiments

Protein–protein interactions involving GPRC5B were assessed using a modified split-TEV assay. A catalytically active mutant of the TEV protease (S219V), which prevents self-cleavage without compromising enzymatic activity, was employed. The canonical TEV protease recognition sequence ENLYFQS (TEVs) was used. The chimeric transcription factor GV, comprising the Gal4 DNA-binding domain and the VP16 transactivation domain (derived from the pM3-VP16 vector; Clontech), was used to drive reporter gene expression.

The TEV protease was split into two fragments: TEV-N (residues 1–118) and TEV-C (residues 119–242). One of the interacting proteins was fused to TEV-N, the TEV substrate site, and GV, whereas the other was fused to TEV-C. Constructs were cloned into the pCDNA6.2/V5-pL Dest vector under the control of the HSV–TK promoter to ensure low expression levels.

HEK293T cells were seeded in 6-well plates and transiently transfected with 3 μg of total DNA per well, comprising 1 μg of each fusion protein (TEV-N and TEV-C), 0.3 μg of the reporter plasmid pNEBr-X1Gluc (New England Biolabs), and 0.2 μg of pCMV-βGal to monitor transfection efficiency and the rest with empty vector. After 48 h, 60 μl of cell culture supernatant was collected, and Gaussia luciferase activity was measured using a CLARIOstar plate reader following the addition of 20 μM native coelenterazine. Luciferase activity was normalized to β-galactosidase activity, determined as previously described. Background signal was defined as the luminescence measured in cells transfected with the GV-fused protein alone.

### BRET experiments

HEK293T cells were transiently transfected with a constant amount (250 ng) of GPRC5B fused to Renilla luciferase and increasing amounts of either GPRC5B-YFP, β-arrestin 2-YFP, or Gα12-YFP. To ensure equal total DNA across conditions, the empty vector pcDNA3.1 was cotransfected as needed. Forty-eight hours post-transfection, cells were washed with PBS, detached, and resuspended in 600 μl of PBS. Aliquots corresponding to 90 μl were distributed in triplicates into a white 96-well plate (catalog no.: 3600; Corning) for BRET measurements and triplicates of 100 μl into a black 96-well plate (catalog no.: 3650; Corning) for fluorescence controls.

BRET was initiated by the addition of h-coelenterazine (Molecular Probes) at a final concentration of 5 μM. Emission signals were recorded using a ClarioSTAR microplate reader (BMG Labtech) with sequential integration at 475 nm (donor emission; bandwidth 445–505 nm) and 530 nm (acceptor emission; bandwidth 500–560 nm). The BRET ratio was calculated as the ratio of light emitted at 530 nm to that at 475 nm, after subtraction of background signals obtained from cells expressing GPRC5B fused to Renilla luciferase alone with pcDNA3.1.

BRET saturation curves were generated by plotting the BRET ratio against the acceptor/donor expression ratio, and data were fitted using nonlinear regression assuming a single binding site model in GraphPad Prism (version 8.00; GraphPad Software, Inc).

### HiBiT assay

HEK293T cells were transiently transfected in 6-well plates with HiBiT-tagged GPRC5B. Where indicated, cotransfection was performed with pCDNA3.1-empty, pCDNA3.1-MLC1, or pCDNA3.1-GlialCAM vectors. After 48 h, cells were harvested in 1 ml of PBS, and 50 μl of the suspension was seeded into white, flat-bottom 96-well plates (Bio-Rad; 50,000 cells/cm^2^). Nanoluciferase activity was measured using Promega reagents following the manufacturer’s instructions. Luminescence was recorded using a CLARIOstar plate reader after 10 min of incubation.

### Primary astrocyte culture and adenoviral transduction

Primary cultures of quiescent astrocytes were prepared from P0 to P2 rat pups as described previously (45). Cultures were maintained in the presence of the mitotic inhibitor cytosine arabinoside (AraC). Adenoviral transduction was performed following established protocols (45). Astrocytes were employed to assess Fyn-dependent signaling, as we observed specific and reproducible activation, justifying their use. In our hands, both HEK293T and HeLa cells showed nonspecific activation of NF-κB -dependent pathways.

### Immunocytofluorescence

Transfected HeLa cells grown in coverslips were fixed in PBS containing 4% paraformaldehyde for 20 min and permeabilized/blocked in PBS with 10% fetal bovine serum and 0.1% Triton X-100 for 1 h at room temperature. Primary antibodies were diluted in blocking buffer and incubated for 1 h at room temperature. Antibodies used included rabbit polyclonal anti-GlialCAM (1:100 dilution; (7)), homemade rabbit anti-GPRC5B (26), mouse anti-FLAG (Sigma), and mouse monoclonal anti-MLC1 (1:100 dilution; (54)). After washing, secondary antibodies were applied for 1 h at room temperature. Coverslips were mounted with Vectashield (Vector Laboratories) containing 1.5 μg/ml 4′,6-diamidino-2-phenylindole and imaged using a DSU spinning disk confocal microscope (Olympus).

Pairs of immunostained cells were manually analyzed to determine whether staining is enriched at cell–cell junctions. To quantify this, we performed intensity profile analyses using ImageJ (http://rsbweb.nih.gov/ij). A straight line is drawn across a pair of cells, and the fluorescence profile along the line is calculated. The cell membranes are identified by the peaks in the profile, and fluorescence intensity is averaged in a region of ±3 pixels around each peak, resulting in F1 and F2 (fluorescence at contact-free membranes of cells 1 and 2) and FC (fluorescence at the cell–cell contact). The relative fluorescence ratio (FR) was defined as FR = FC/(F1 + F2). Cells with FR >1 were considered to have junctional enrichment of the immunolabeled protein. This criterion has been used to quantify the percentage of cells showing junctional localization. This method has been consistently applied in our previous studies and has proven to be a reliable approach to assess subcellular localization.

### Western blotting

HEK293T and astrocyte lysates were prepared as described previously (45). Equal protein loading was confirmed by probing for tubulin (mouse monoclonal antitubulin; Millipore). For Western blot detection of GPRC5B–LgBiT fusion proteins, we used the commercially available rabbit anti-GPRC5B (HPA015247; Sigma), detecting nearly all the C termini of the protein, since our antibody does not detect the fusion protein, likely because its epitope lies at the end of the C-terminal region, which may be masked in the fusion construct. For endoglycosidase treatments, cell lysates were incubated with endo H or endo F enzymes according to the manufacturer’s instructions.

#### Surface biotinylation assay

HEK293T cells were transiently transfected in 6-well plates. After 48 h, cells were placed on ice for 15 min and washed twice with PBS supplemented with 0.5 mM CaCl_2_ and 1 mM MgCl_2_ (PBS–CM). Cells were then incubated with PBS–CM containing 1 mg/ml biotin for 30 min at 4 °C on a shaker. Subsequently, cells were washed twice with PBS–CM containing 20 mM lysine for 5 min, followed by two additional washes with PBS–CM. At this point, cells were lysed in radioimmunoprecipitation assay buffer supplemented with protease inhibitors for 1 h at 4 °C, and lysates were centrifuged for 15 min at maximum speed. The supernatant was collected and incubated with streptavidin bead suspension for 60 min at 4 °C. Samples were then centrifuged for 3 min at 4000*g*, and the supernatant was collected. Beads were washed four times with radioimmunoprecipitation assay buffer and eluted in SDS sample buffer containing 5% β-mercaptoethanol to obtain the surface (biotinylated) protein fraction.

### Statistics

All quantitative data are presented as mean ± SD. Statistical significance was determined using GraphPad Prism. For comparison between two groups, a two-tailed unpaired Student’s *t* test was used. For multiple group comparisons, one-way ANOVA followed by Tukey‘s post hoc test was applied. The statistical test used in each case is indicated in the respective figure legend.

## Data availability

The datasets generated during the current study are available from the corresponding author on reasonable request.

## Supporting information

This article contains supporting information.

## Conflict of interests

The authors declare that they have no conflicts of interest with the contents of this article.
