# Supplementary material for: Regulation of the orphan G-protein–coupled receptor GPRC5B by MLC1 and the cell adhesion molecule GlialCAM in megalencephalic leukoencephalopathy
Source: J Biol Chem. 2025 Nov 27;302(1):110987. doi: 10.1016/j.jbc.2025.110987 (PMC12775950; doi:10.1016/j.jbc.2025.110987)
Supplement: Supplementary Material 1 [file mmc1.pdf]

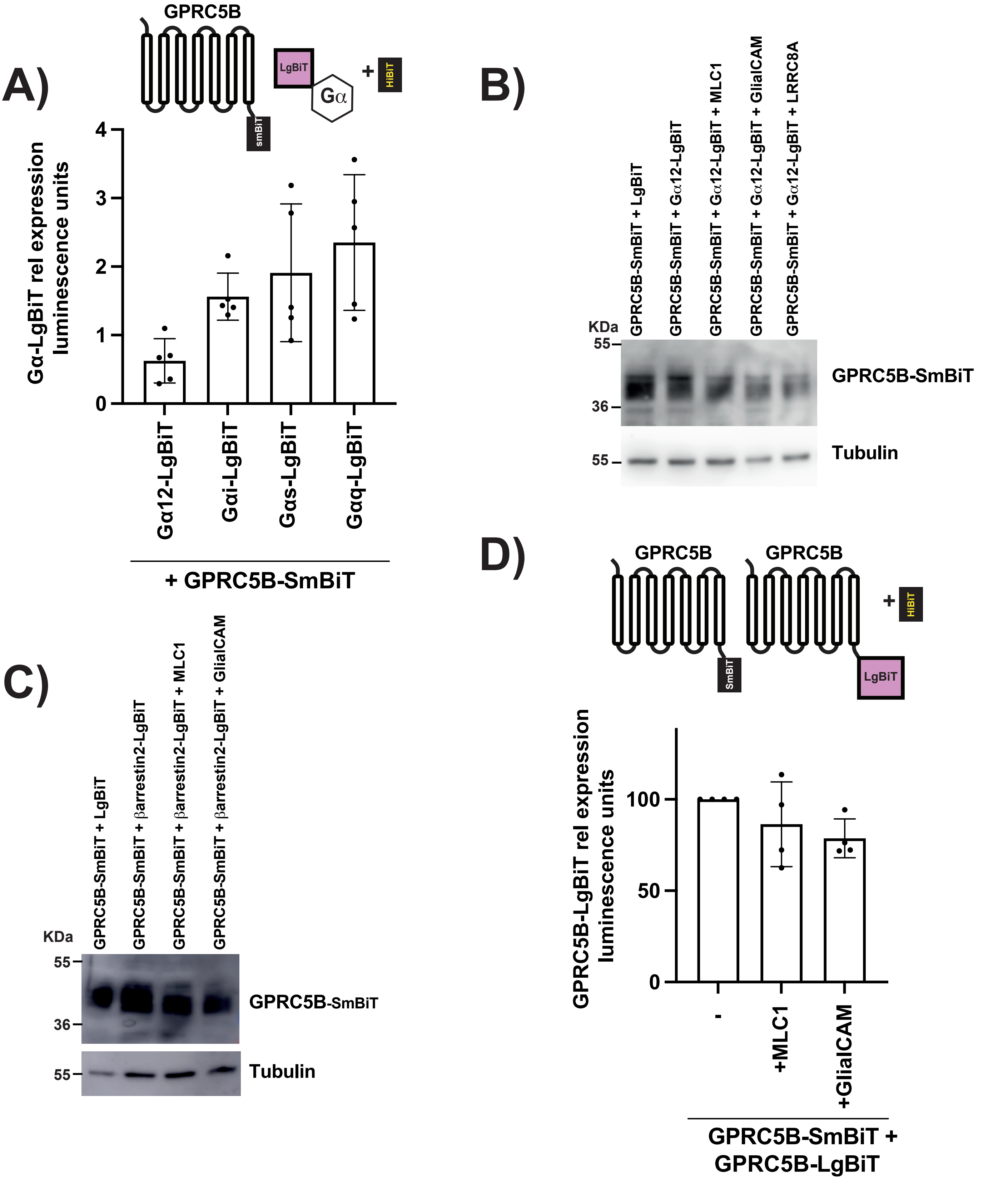

Supplementary Figure 1. Control experiments for NanoBiT-based signaling assays. **(A)** Schematic representation and quantification of mini-G protein expression levels in HEK293T cells transfected with GPRC5B-SmBiT and mini-Gα subunits fused to LgBiT (Gαs, Gαi, Gαq, Gα12). Expression was measured using the Nano-Glo HiBiT Lysis Detection System with HiBiT complementation. Despite lower expression of Gα12 compared to other subunits, GPRC5B showed the strongest NanoBiT signal with Gα12 (see Fig. 2B), indicating robust interaction independent of expression level. **(B)** Western blot analysis of GPRC5B-SmBiT expression in HEK293T cells co-transfected with Gα12-LgBiT and either MLC1, GlialCAM, or LRRC8A. Tubulin was used as a loading control. Comparable GPRC5B expression across conditions confirms that changes in NanoBiT signal reflect true interaction differences. **(C)** Western blot analysis of GPRC5B-SmBiT expression in cells co-transfected with β-arrestin2-LgBiT and either MLC1 or GlialCAM. Tubulin served as a loading control. GPRC5B levels remained consistent across conditions, supporting the interpretation of differential β-arrestin2 recruitment shown in Fig. 3B. **(D)** Schematic and quantification of GPRC5B-LgBiT expression in oligomerization assays. HEK293T cells were co-transfected with GPRC5B-SmBiT and GPRC5B-LgBiT, with or without MLC1 or GlialCAM. Expression was measured using HiBiT complementation, using the Nano-Glo HiBiT Lysis Detection System. Similar GPRC5B-LgBiT levels across conditions confirm that changes in NanoBiT signal reflect modulation of receptor dimerization rather than expression differences.

SupFigure 1_Resub
